# Single-Center Experiences: A Comparison of Intracorporeal and Extracorporeal Anastomosis Outcomes in Right Hemicolectomy

**DOI:** 10.7759/cureus.59339

**Published:** 2024-04-30

**Authors:** Audrey Kim, Munyaradzi G Nyandoro, Linda Vu, Ruben Rajan, Abraham Jacob

**Affiliations:** 1 General Surgery, Royal Perth Hospital, Perth, AUS; 2 General and Colorectal Surgery, Royal Perth Hospital, Perth, AUS

**Keywords:** intracorporeal, extracorporeal, bowel function, anastomosis, hemicolectomy

## Abstract

Background

Anastomosis formed in minimally invasive laparoscopic right hemicolectomy (LRH) may be achieved intra-corporeally (ICA) or extra-corporeally (ECA). This study compared the return of bowel function and other associated early patient outcomes and morbidity rates after an ICA or ECA in LRH.

Methodology

The study conducted a single-center retrospective cohort study of elective LRH from January 2021 to September 2023. Patient demographics, surgical techniques, and outcomes were analyzed using IBM SPSS Statistics for Windows, Version 29.0 (IBM Corp., Armonk, NY).

Results

Ninety participants underwent LRH, and the anastomotic type was evenly distributed - with male patients comprising 53 (58.9%) of the total. The mean age was 64 (standard deviation [SD] ±16.8) years, and the median body mass index (BMI) was 27.0 (interquartile range [IQR] = 7.8). The mean follow-up period was 5.1 (SD ± 6.0) months.

Univariate analysis showed that ICA had a shorter time for return of bowel function (*P* < 0.01). Additionally, ICA was associated with lower pain scores (*P* < 0.01), low morbidity (*P* = 0.02), and shorter hospital stays (*P* = 0.01). When comparing ICA to ECA, no significant difference was observed for procedure duration (*P* = 0.13), anastomotic leak (AL, *P* = 1.00), surgical-site infections (*P* = 0.36), lymph node yield (*P* = 0.26), and any-cause mortality.

Multivariate logistic regression, controlling for statistically insignificant confounding factors, revealed that ECA was significantly and independently associated with increased time to first flatus (odds ratio [OR] 2.3, *P* = 0.01) and higher average postoperative pain (OR 1.5, *P* = 0.02) compared to ICA.

Conclusions

This single-center experience showed that ICA is associated with a quicker return to normal bowel function and low morbidity outcomes. ICA participants were positively associated with clinically relevant and health economics outcomes of shorter hospital stays without significantly adding to the procedure's duration times or compromising principles of oncological resection yield.

## Introduction

Techniques for right hemicolectomy have evolved over the years, from the first descriptions by Reybard in 1932 to the modern-day practice of laparoscopic operative approaches for intraabdominal pathologies. The Clinical Outcomes of Surgical Therapy (COST) trial validated minimally invasive colectomy surgeries by addressing initial concerns of inadequate oncologic resection margins and trocar site-related recurrences [1,2]. Furthermore, the literature has shown laparoscopic surgery to be associated with reduced morbidity, less postoperative pain, and shorter length of hospital stay (LoHS) [1-4].

In the era of laparoscopic surgery, the techniques for anastomosis formation have evolved, and there is currently no standardized technique and limited data on specific outcomes of right hemicolectomies. The anastomosis can be achieved either intracorporeally (ICA) or extracorporeally (ECA), stapled or hand sewn, and many orientations can be performed (side-to-side, end-to-end, end-to-side, and in an iso-peristaltic or anti-peristaltic fashion). Rajan et al. reported that stapled end-to-side anastomosis had the lowest rate of anastomotic leaks (ALs) [5].

Intracorporeal anastomosis (ICA) is less invasive than extracorporeal anastomosis (ECA) and is associated with shorter LoHS and reduced short-term morbidity [6]. The difference in early postoperative complications when comparing ICA to ECA may partly be explained by the larger incision required for ECA to extract the bowel and perform the anastomosis. There is conflicting evidence for the benefits of ICA compared to ECA regarding early postoperative complications [6]. Weeks et al. [7] demonstrated that the ICA had less postoperative pain and shorter time to flatus; however, Jamali et al. [8] found that ECA had a shorter operation duration and better oncological outcomes. In contrast, a long-term follow-up meta-analysis by Aiolfi et al. [9] found no significant differences in long-term survival outcomes in both groups.

This paper describes a single-center early state-wide adoption experience in Western Australia, comparing ICA and ECA surgical approaches and their associated short-term outcomes. The primary aim was to determine the time interval for bowel function to return after each procedure (ICA vs. ECA).

## Materials and methods

A retrospective analysis of a prospectively maintained database was performed on all adult patients who underwent a laparoscopic right hemicolectomy from January 2021 to September 2023 at a colorectal surgery unit in a tertiary hospital in Western Australia. Relevant clinicopathological data, technical surgical indicators, and clinical outcomes were collected from medical records, radiology, and pathology reports. Two investigators (AK and MN) independently recorded these data to ensure accuracy. 

Primary outcome 

The return of bowel function was defined as either time to first flatus or bowel motion.

Secondary outcome 

Anastomotic leak (AL) was defined as radiological evidence for AL, intervention in the form of radiological drainage of perianastomotic collection, or evidence of AL at the return to the theater [4]. Anastomotic bleed (AB) was defined as a postoperative AB resulting in transfusion, endoscopic intervention, or return to theatre. Wells et al. [10] defined postoperative ileus (POI) as an interval period between surgery and recovery where there is a disturbance in the gastrointestinal (GI) function. This can be categorized as obligatory POI - an early period of ileus, usually mild and self-limiting, occurring in all patients undergoing surgery, the duration of which is dependent on the type of surgery and approach and comorbidities. Prolonged POI is the pathological extension characterized by persistent ileus beyond the *obligatory POI *period. It is associated with more severe symptoms with a consensus diagnosis at post-op day 4 [10]. SSI was defined as superficial or deep space infection with associated clinical, biochemical, microbiological, and imaging correlation. Incisional hernia was diagnosed either clinically or on radiological imaging (computed tomography or ultrasound). Postoperative pain was recorded as the average pain score on the first day post-operation and the average pain score on the day of discharge. The Clavien-Dindo classification was used to categorize the complications. Operative time was defined as the time in minutes between the initial incision and closure of the surgical wound. Thirty-day representation and readmission rates were recorded. Overall survival was determined by the date of death recorded or at the date of the last follow-up.

Procedure description

Expertise

The procedures were performed at a tertiary teaching referral center for minimally invasive colorectal surgery staffed by high-volume, Colorectal Surgical Society of Australia and New Zealand (CSSANZ) board-accredited colorectal specialists comprising two early state-wide adopters of ICA and two traditional ECA surgeons.

Patient Preparation and Position

The patients were supine, and the modified Llyod Davies position was used for Crohn’s, extended right, or emergency cases.

Port Placement

Peritoneal entry was gained with either a 5 mm left upper quadrant (LUQ) optical entry or standard Hasson’s umbilical port. The remaining ports were placed under vision in a conventional right hemicolectomy triangulation, and three 5 mm ports were placed in the left iliac fossa, suprapubic, and epigastrium. The options for stapling port placement were either a 10 mm suprapubic or a 12 mm LIF port, which was extended and used for specimen extraction as a Pfannenstiel incision or a left lower quadrant incision. Some cases had a midline extraction.

Colon Mobilization

The authors preferred medial to lateral dissection with infra ileocolic-duodenum mobilization off the mesocolon.

Vascular Control

The ileocolic pedicle was taken high at the superior mesenteric artery (SMA) and ileocolic artery junction. Hemlocks (5 mm) were deployed individually to ligate the veins and arteries. The line of Toldt was then incised to complete mobilization.

Resection

Mesentery to the transverse colon was divided first, followed by the small bowel, allowing resection to vascular demarcation before division using EndoGIA 60 Tan.

Anastomosis

A 3-0 PDS crotch stitch was placed to offset the tension and for orientation. An enterotomy and a colotomy were performed with a Raytec placed underneath for spillage control. A side-to-side stapled isoperistaltic anastomosis was created using an Endo GIA 60 mm Tan cartridge. The entero-colotomy was closed in two layers with 3-0 PDS Stratafix.

Extraction

The specimen was extracted from a small Pfannenstiel incision or an extended LIF incision with wound protection using a small, capped Alexis.

Statistical analysis 

Baseline characteristics and outcome data for each procedure group were described using mean (± standard deviation [SD]), median (interquartile range [IQR]), or frequencies/proportions (%), depending on the distribution. The nonparametric Mann-Whitney U test analyzed outcomes for continuous unpaired variables. Dichotomous results were compared between groups using *χ*^2^ or Fisher’s exact tests with no adjustment for multiple comparisons. The univariate and multivariate analysis evaluated the relationship between the overall complication risk and other factors. Multivariate binary logistic regression was used to model the type of anastomosis outcomes while adjusting for potential confounders about patient factors (age, obesity), functional factors (return of bowel function), and pathological (benign vs. malignant). The independent variables selected in the regression model were consistent with the current literature on outcome factors. All analyses were performed using IBM SPSS Statistics for Windows, Version 29.0 (IBM Corp., Armonk, NY), and a two-tailed *P*-value < 0.05 was considered statistically significant.

Permissions

The Royal Perth Hospital Group Quality Improvement Committee approved ethics through the low-risk alternate pathway (Quality Activity Ref: 47322).

## Results

Patient characteristics 

Ninety participants underwent a laparoscopic right hemicolectomy during the study period. The anastomosis type was evenly distributed; the primary indication was malignancy (*n* = 79, 87.8%). Most participants were male (*n* = 53, 58.9%), with a mean age of 65 (SD ±16.8) years, and the mean overall LoHS was five (SD ±4) days. The median BMI was 27.0 (IQR = 7.8), a third of the participants were obese (*n* = 29, 32.2%). Almost half of the participants had multiple risk factors for surgical site infections (*n* = 44, 48.9%). However, most of the patients were not smokers (*n* = 76, 84.4%), did not have diabetes (*n* = 72, 80.0%), nor were they classified as immunosuppressed (*n* = 80, 88.9%) (Table 1).

**Table 1:** Summary of participant characteristics. Values are the number of participants (%) unless otherwise indicated. ^a^IQR = interquartile range, M = mean, SD = standard deviation, *n* = number. ^b^IVAB, intravenous antibiotic. ^c^ICA and ECA = intracorporeal and extracorporal anastomoses. ^d^PDS, polydioxanone suture

Variable (*N* = 90)		Number (*n*), proportion (%)
Demographic characteristics^a^	Male: Female, *n* (%)	53 (58.9): 37 (41.1)
	Age (years: mean ± SD)	64.6 ± 16.8
	BMI (number: median, IQR)	27.0 (7.8)
	Length of hospital (days: median, IQR)	5.0 (4.0)
	Clinic follow-up (months: mean ± SD)	5.1 ± 6.0
Comorbidities	Normal albumin, *n* (%)	77 (85.6)
	Hypertension, *n* (%)	49 (54.4)
	More than one risk factor, *n* (%)	44 (48.9)
	Obese, *n* (%)	29 (32.2)
	Respiratory disease (COPD), *n* (%)	21 (23.3)
	Coronary artery disease, *n* (%)	20 (22.2)
	Diabetes, *n* (%)	18 (20.0)
	Smoker, *n* (%)	14 (15.6)
	Immunotherapy, *n* (%)	6 (6.7)
	Steroid therapy, *n* (%)	4 (4.4)
Operation characteristics	IVABs on induction^b^ *n* (%)	90 (100)
	Mechanical bowel-prep, *n* (%)	81 (90.0)
	Elective admission, *n* (%)	78 (86.7)
	Consultant-led operation, *n* (%)	70 (77.8)
Indication for surgery	Malignancy, *n* (%)	79 (87.8)
	Benign pathology, *n* (%)	11 (12.2)
Type of skin-prep	Povidone/Iodine 10%, *n* (%)	45 (50.0)
	Chlorhexidine 2%, *n* (%)	45 (50.0)
Type of anastomosis^c^	ICA, *n* (%)	45 (50.0)
	ECA, *n* (%)	45 (50.0)
Specimen extraction site	Periumbilical, *n* (%)	56 (62.2)
	Pfannenstiel, *n* (%)	17 (18.9)
	Left lower quadrant, *n* (%)	17 (18.9)
Type of fascia closure	PDS^d^, *n* (%)	86 (95.6)
	Other, *n* (%)	4 (4.4)
Wound protector	Wound protector used, *n* (%)	83 (92.2)
	No wound protector, *n* (%)	7 (7.8)

Categorical variables of participant characteristics 

Baseline patient demographics were similar between the subgroups of ICA and ECA. Univariate analysis showed significant positive associations between the type of anastomotic approach and specimen extraction site, with the ICA extraction site preferentially being an off-midline while the ECA was in the midline. In addition, there is also a significant positive association with surgeon experience, with the majority of ICA being performed by the consultant and ECA being done almost equally between consultants and trainees (Table 2).

**Table 2:** Characteristics of laparoscopic right hemicolectomy patients and univariate chi-square (Pearson chi-square analysis, and Fisher’s exact test [cell values <5]) results for independent variables, with the type of anastomosis as the dependent variable. Values are the number of participants (%) unless otherwise indicated. Bold values denote significance at *P* < 0.05. ^a^ASA, American Association of Anesthesiology (score). ^b^SSI, surgical site infection. ^c^IVABs, intravenous antibiotics. ^d^COPD, chronic obstructive pulmonary disease.

Variable (*N* = 90)	Total cohort	Intracorporeal anastomosis			Extracorporeal anastomosis		
	*n* (%)	n	%	*P*-value	n	%	*P*-value
Gender							
Male	53 (58.9)	25	47.2	0.520	28	52.8	0.520
Female	37 (41.1)	20	54.1		17	45.9	
Age (years)							
60 years and younger	29 (32.2)	15	51.7	0.822	14	48.3	0.822
61 years and older	61 (67.8)	30	49.2		31	50.8	
Weight categories							
Healthy weight	28 (31.1)	14	50.0	1.000	14	50.0	1.000
Overweight/Obese	62 (68.9)	31	50.0		31	50.0	
ASA score^a^							
One	6 (6.7)	2	33.3	0.356	4	66.7	0.356
Two	42 (46.7)	19	45.2		23	54.8	
Three	41 (45.6)	24	58.5		17	41.5	
Four	1 (1.1)	0	0.0		1	100	
Specimen extraction site^b^							
Midline extraction	56 (62.2)	12	21.4	<0.001	44	78.6	<0.001
Off midline	34 (37.8)	33	97.1		1	2.9	
Operative time							
120 minutes or less	24 (26.7)	10	41.7	0.340	14	58.3	0.340
More than 121 minutes	66 (73.3)	35	53.0		31	47.0	
Admission type							
Elective	78 (86.7)	38	48.7	0.535	40	51.3	0.535
Emergency	12 (13.3)	7	58.3		5	41.7	
IVABs on induction^c^							
Yes	90 (100)	45	50.0	-	45	50.0	-
Skin-prep							
Povidone/Iodine	45 (50.0)	20	44.4	0.292	25	55.6	0.292
Chlorhexidine	45 (50.0)	25	55.6		20	44.4	
Primary surgery							
Malignancy	79 (87.8)	42	53.2	0.108	37	46.8	0.108
Benign pathology	11 (12.2)	3	27.3		8	72.7	
Estimated blood loss							
Minimum (<10 mL)	80 (88.9)	41	51.2	0.739	39	48.8	0.739
More than 11 mL	10 (11.1)	4	40.0		6	60.0	
Surgeon experience							
Consultant	70 (77.8)	44	62.9	<0.001	26	37.1	<0.001
Trainee	20 (22.2)	1	5.0		19	95.0	
Mechanical bowel-prep							
No	9 (10.0)	2	22.2	0.157	7	77.8	0.157
Yes	81 (90.0)	43	53.1		38	46.9	
Hypertension							
No	41 (45.6)	20	48.8	0.832	21	51.2	0.832
Yes	49 (54.4)	25	51.0		24	49.0	
Respiratory (COPD)^d^							
No	69 (76.7)	38	55.1	0.081	31	44.9	0.081
Yes	21 (23.3)	7	33.3		14	66.7	
Smoker							
No	76 (84.4)	41	53.9	0.144	35	46.1	0.144
Yes	14 (15.6)	4	28.6		10	71.4	
Diabetes							
No	72 (80.0)	37	51.4	0.598	35	48.6	0.598
Yes	18 (20.0)	8	44.4		10	55.6	
More than one risk factor							
No	46 (51.1)	22	47.8	0.673	24	52.2	0.673
Yes	44 (48.9)	23	52.3		21	47.7	

Continuous variables of participant characteristics 

Patient demographics of age, ASA, and BMI were evenly distributed. There was a statistically significant difference in time to first flatus 1.5 days vs. 3.2 days (*P* < 0.001) and first bowel motion 2.3 days vs. 4.2 days (*P* < 0.001) between ICA and ECA, with ICA having a quicker return to bowel function. In addition, ICA was also significantly associated with less postoperative pain on day 1 (*P* < 0.001) and day of discharge (*P* = 0.008). The ICA had less severe complications per the Clavien-Dindo Classification 1.4 vs. 2.0 (*P* = 0.023). The ICA participants had a shorter initial LoHS of 5.4 vs. 7.5 days (*P* = 0.009). While the time interval to all-cause mortality for the two subgroups was trending, it did not reach statistical significance. Furthermore, no statistical difference was noted for procedure duration, the time interval to surgical site occurrences, lymph node harvest, LoHS on representation, and time interval to represent post-discharge (Table 3).

**Table 3:** Comparison of continuous characteristics for patients with laparoscopic right hemicolectomy. Values are the number of participants (%) unless otherwise indicated. Nonparametric independent-samples Mann Whitney U test. Bold values denote significance at *P* < 0.05. ^a^BMI, body mass index. ^b^ASA, American Association of Anesthesiology (score). ^c^LoHS, length of hospital stay. ^d^Lymph yield: cancer cases only. ^e^ SSO, surgical site occurrence (infection/dehiscence/seroma/hematoma). ^f^OPC, outpatient clinic. ICA, intracorporeal anastomosis; ECA, extracorporal anastomosis

Variable (*N* = 90)	Anastomosis subtype		
	ICA	ECA	*P*-value
Age (years)	65.0	64.2	0.738
BMI (nn)^a^	29.3	27.3	0.277
ASA (nn)^b^	2.5	2.3	0.214
Initial LoHS (days)^c^	5.4	7.5	0.009
Procedure duration (minutes)	158	143	0.129
Lymph node harvest (nn)^d^	17.7	16.3	0.261
Time to first flatus (days)	1.5	3.2	<0.001
Time to first bowel motion (days)	2.3	4.2	<0.001
Average pain score day 1 post-op (nn)	1.5	3.3	<0.001
Average pain score day of discharge (nn)	0.4	1.1	0.008
Clavien-Dindo classification	1.4	2.0	0.023
Time interval to surgical site occurrences (days)^e^	9.0	4.3	0.400
Total OPC appointments (nn)^f^	1.9	3.5	<0.001
Clinic follow-up (days)	79.4	264	<0.001
Clinic follow-up (weeks)	11.0	37.4	<0.001
Clinic follow-up (months)	2.1	8.2	<0.001
Time interval to represent post-discharge	5.5	6.0	1.000
LoHS representation (days)^c^	3.0	1.5	0.381
Time interval to all-cause mortality (months)	5.0	21.8	0.057

Complication profile by type of anastomosis

Univariate analysis showed significant associations between the overall complication risk and the type of surgical approaches. ICA was significantly associated with a lower risk of paralytic ileus (3.3% vs. 18.9%, *P* < 0.001), reduced use of prokinetics (7.8% vs. 24.4%, *P* < 0.001), and decreased requirement for aperients (4.4% vs. 21.2%, *P* < 0.001). In addition, ICA was also significantly associated with a reduced risk of requiring post-op nutritional supplementation (*P* = 0.039). The overall post-op complication rate was lower for ICA, 14.4% vs. 28.9% (*P* = 0.006), and the CD category status was also lower for ICA compared to ECA (*P* = 0.045). However, no significant differences were detected for anastomotic leak, SSI, wound dehiscence, incisional hernia, or readmission rates between the two surgical approaches (Table 4).

**Table 4:** Complications profile characteristics of laparoscopic right hemicolectomy participants and univariate chi-square (Pearson chi-square analysis, and Fisher’s exact test [for cell values <5]) results for independent variables, with the type of anastomosis as the dependent variable. Values are the number of participants (%) unless otherwise indicated. Bold values denote significance at *P *< 0.05. ^a^30 days post initial surgery. ^b^More than 30 days post-surgery. ^c^Representation other than for routine clinic follow-up. ^d^Ileus diagnosed clinically and on imaging defined. ^e^PPN, peripheral parenteral nutrition. ^f^TPN, total parenteral nutrition. ^g^SSI, surgical site infection.

Variable (*N* = 90)	Total cohort	Intracorporeal anastomosis			Extracorporeal anastomosis		
	*n* (%)	n	%	*P*-value	n	%	*P*-value
Anytime post-op complication							
No	51 (56.7)	32	35.6	0.006	19	21.1	0.006
Yes	39 (43.3)	13	14.4		26	28.9	
Any inpatient complication							
No	57 (63.3)	36	40.0	0.001	21	23.3	0.001
Yes	33 (36.7)	9	10.0		24	26.7	
Any outpatient complication							
No	76 (84.4)	39	43.3	0.561	37	41.1	0.561
Yes	14 (15.6)	6	6.7		8	8.9	
Clavien-Dindo classification							
One	19 (38.0)	12	24.0	0.045	7	14.0	0.045
Two	29 (58.0)	10	20.0		19	38.0	
Three	2 (4.0)	0	0.0		2	4.0	
Any 30-day post-op complication ^a^							
No	55 (61.1)	35	38.9	0.001	20	22.2	0.001
Yes	35 (38.9)	10	11.1		25	27.8	
Any delayed complication ^b^							
No	88 (97.8)	45	50.0	0.494	43	47.8	0.494
Yes	2 (2.2)	0	0.0		2	2.2	
Represented first month ^c^							
No	80 (88.9)	40	44.4	1.000	40	44.4	1.000
Yes	10 (11.1)	5	5.6		5	5.6	
Readmitted first month							
No	83 (92.2)	40	44.4	0.434	43	47.8	0.434
Yes	7 (7.8)	5	5.6		2	2.2	
Electrolyte disturbances							
No	62 (68.9)	31	34.4	1.000	31	34.4	1.000
Yes	28 (31.1)	14	15.6		14	15.6	
Paralytic ileus ^d^							
No	70 (77.8)	42	46.7	<0.001	28	31.1	<0.001
Yes	20 (22.2)	3	3.3		17	18.9	
Required prokinetics							
No	61 (67.8)	38	42.2	0.023	23	25.6	0.023
Yes	29 (32.2)	7	7.8		22	24.4	
Required PPN ^e^							
No	76 (84.4)	42	46.7	0.039	34	37.8	0.039
Yes	14 (15.6)	3	3.3		11	12.2	
Required TPN ^f^							
No	81 (90.0)	43	47.8	0.157	38	42.2	0.157
Yes	9 (10.0)	2	2.2		7	7.8	
Required aperients							
No	67 (74.4)	41	45.6	<0.001	25	28.9	<0.001
Yes	23 (25.6)	4	4.4		19	21.2	
Anytime anastomotic leak							
No	88 (97.8)	44	48.9	1.000	44	48.9	1.000
Yes	2 (2.2)	1	1.1		1	1.1	
Anytime SSI ^g^							
No	80 (88.9)	43	47.8	0.090	37	41.1	0.090
Yes	10 (11.1)	2	2.2		8	8.9	
Anytime wound dehiscence							
No	85 (94.4)	44	48.9	0.361	41	45.6	0.361
Yes	5 (5.6)	1	1.1		4	4.4	
Anytime incisional hernia							
No	89 (98.9)	45	50.0	1.000	44	48.9	1.000
Yes	1 (1.1)	0	0.0		1	1.1	

Summary of all cohort postoperative complications 

The overall complication rate or cohort morbidity was 43.3% (*n* = 39), of which paralytic ileus was the most common morbidity reported (*n* = 21, 23.3%), followed by SSI (*n* = 10, 11.1%). Most complications were low-order CD category (*n* = 48, 96%). Other noteworthy complications reported were lower GI bleed (6.7%), surgical wound dehiscence (5.6%), bowel obstruction (4.4%), and an anastomotic leak rate of 2.2%. No sentinel deaths were directly related to the surgical procedure, but the overall all-cause mortality during the follow-up period was 7.8% (*n* = 7) (Table 5).

**Table 5:** Summary of postoperative complications - combined (inpatient and outpatient). Values are the number of participants (%) unless otherwise indicated. ^a^Delayed - representation beyond the initial 30-day follow-up. ^b^Sentinel death - defined as death occurring within 30 days post-op and directly attributable to the surgery.

Variable (*N* = 90)		Number (Proportion), *n* (%)
Postoperative outcomes	Any post-op complication	39 (43.3)
	Any 30-day complication	35 (38.9)
	Paralytic ileus	21 (23.3)
	Surgical site infection	10 (11.1)
	Any-cause mortality	7 (7.8)
	Lower gastrointestinal bleed	6 (6.7)
	Surgical site wound dehiscence	5 (5.6)
	Intestinal obstruction	4 (4.4)
	Anastomotic leak	2 (2.2)
	Unplanned return to theater	1 (1.1)
	Incisional hernia (delayed) ^a^	1 (1.1)
	30-day representation	10 (11.1)
	30-day readmission	7 (7.8)
	Delayed representation^a^	2 (2.2)
	Sentinel 30-day mortality^b^	0 (0.0)
	Clavien-Dindo classification	
	I	19 (38.0)
	II	29 (58.0)
	III	2 (4.0)

Regression analysis

Multivariate logistic regression analysis showed that in comparison to ICA, ECA was significantly and independently associated with increased time to first flatus (odds ratio [OR] 2.3, 95% confidence interval [CI] 1.2-4.3, *P* = 0.012) and higher average postoperative pain (OR 1.5, 95% CI 1.1-2.2, *P* = 0.020). The regression model controlled for the potential confounders and univariate significant factors of age, BMI, initial LoHS, gender, any post-op complications, paralytic ileus, postoperative - PPN, prokinetics, and aperients (Table 6).

**Table 6:** Multivariate logistic regression, the likelihood of an extra-corporeal anastomosis in comparison to intracorporeal anastomosis as the dependent variable (all participants, N = 90). Bold values denote significance at *P *< 0.05. ^a^Age, body mass index (BMI), initial length of hospital (LoHS), time to first flatus, and average day-1 pain score are continuous variables. ^b^This parameter is compared to male, which is set to zero. ^c^This parameter is compared to no post-op compilations, which is set to zero. ^d^This parameter is compared to no paralytic ileus, which is set to zero. ^e^This parameter is compared to requiring post-op peripheral parenteral nutrition (PPN), which is set to zero. ^f^This parameter is compared to requiring post-op prokinetics, which is set to zero. ^g^This parameter is compared to not requiring post-op aperient, which is set to zero.

Covariates in the model		Anastomosis type as the dependent variable			
		*P*-value	OR	95% CI	
				Lower	Upper
Age ^a^	Years	0.427	1.015	0.98	1.05
BMI ^a^	n	0.615	0.973	0.88	1.08
Initial LoHS ^a^	Days	0.832	1.022	0.84	1.25
Time to first flatus ^a^	Days	0.012*	2.262	1.20	4.26
Average day 1 post-op pain score (0-10)	Pain score	0.020*	1.538	1.07	2.21
Gender ^b^	Female	0.301	1.898	0.56	6.40
Any post-op complication ^c^	Yes	0.805	1.189	0.30	4.70
Paralytic ileus ^d^	Yes	0.458	2.385	0.24	23.7
Required PPN ^e^	Yes	0.523	2.425	0.16	36.9
Required prokinetics ^f^	Yes	0.935	1.072	0.20	5.66
Required aperients ^g^	Yes	0.107	3.774	0.75	19.0

## Discussion

Minimally invasive laparoscopic colectomies are now the preferred standard for managing benign and malignant colonic diseases [1,2]. ICA and ECA are the two anastomotic technical approaches described in the literature for restoring intestinal continuity in minimally invasive right hemicolectomies [3,4]. ICA is reportedly a more technically challenging anastomosis; therefore, advanced training in this skill set is required for its safe execution and to ensure good patient outcomes with low morbidity and mortality [2-4,6-8]. Although technically challenging, various studies have shown improved postoperative outcomes and fewer complications associated with ICA compared to ECA [3,4,6,8,9,11-13]. In this retrospective study comparing early outcomes of ICA in comparison to ECA in laparoscopic right hemicolectomy, the study observed a significantly quicker recovery of bowel function with a shorter time to first flatus and first bowel motion, along with less overall morbidity associated with ICA. 

Bowel function recovery 

This study demonstrated that participants who underwent ICA were associated with shorter times to first flatus and first bowel motion, consistent with various meta-analyses and systematic reviews [3,4,9,11-14]. It is thought that the reduced bowel handling and mobilization required for ICA, when compared to ECA, results in a shorter time to regain bowel function. A meta-analysis conducted by both Emile et al. [12] and Aiolfi et al. [9] suggested that decreased bowel manipulation translates to reduced bowel traction and a lower risk of mesentery twisting. Similarly, a randomized controlled study performed by Mari et al. [15] further explained this phenomenon by suggesting that there is less surgical stress response from the bowel, as evidenced by the lower inflammatory markers postoperatively in ICA when compared to ECA. This study postulates that surgical stress burden is reduced in the formation of ICA due to minimal tumor manipulation and reduced bowel manipulation because the specimen is only retrieved once the anastomosis has been completed. 

In contrast, historically, Ricci et al. [16] and Wu et al. [4] showed no significant difference in time to first flatus between ICA and ECA subgroups. Frigault et al. [17] found no significant difference in time to the first bowel movement and reported a prolonged time to flatus for ICA participants. These contradictory results can be attributed to the challenges of early technique adoption, such as the steep learning curve due to the complexity of the ICA and the relatively longer operative duration. However, these findings are primarily historical, with most recent studies agreeing with this study's findings.

Morbidity and postoperative complications

In this study, participants who underwent an ICA had a lower morbidity rate, as reflected by a significantly lower CD category. A higher incidence of SSI was noted in the ECA group. However, this did not reach statistical significance, which is consistent with the meta-analysis by Wu et al. [4]. Furthermore, this study’s findings of increased SSI risk for ECA are consistent with other published literature [9,16]. Ricci et al. [16] and Martinek et al. [18] reported significantly higher rates of developing SSI and rate of incisional hernia in the ECA group compared to ICA. The observed increased risk profile for ECA can be attributed to likely contamination of the incisional wound during exteriorization of the colon when performing the anastomosis [16,18]. Aiolfi et al. [9] reported a 50% risk reduction in SSI rates in ICA compared to ECA. They suggested that bowel extraction in ECA is associated with increased tissue and bowel trauma, which poses a higher degree of risk of contamination to surrounding soft tissue despite the usage of wound protectors. In this study, most ECA participants had a midline extraction site compared to the off-midline extraction site utilized for the ICA. A midline extraction site is associated with an increased risk of incisional hernia [4], which will be a question for the long-term follow-up study. 

Performing laparoscopic anastomosis is regarded as technically challenging, given the restraints of both a confined space and a narrow visualization field. This was previously associated with an increased risk of anastomotic leak [14,19]. A meta-analysis by Emile et al. [12] and Carnuccio et al. [14] demonstrated a significantly higher rate of anastomotic leak in the ICA group versus the ECA. In contrast, this study showed a similar anastomotic leak rate between the two groups. This observation is in keeping with multiple other studies that also showed identical incidence rates [9] or no differences in leak rates when comparing ICA and ECA [4,13,16]. Furthermore, several studies have reported minimal or similar risk profiles for anastomosis leak rates, bleeding incidence, and postsurgical intra-abdominal abscess formation between the two surgical approaches of ICA and ECA [17,20-23]. The other reason for the observed low leak rate in the ICA population can be attributed to surgeon expertise level, as this procedure was performed at a significantly higher rate by CSSANZ fellowship-trained consultant surgeons. 

Continuous characteristics (operative time, hospital stay, and lymph node yield)

As previously reported in the literature, one of the main drawbacks of ICA is the associated prolonged operative duration due to the steep learning curve [11,17,21,24-27]. This study, however, did not observe a significant difference in procedure duration times between ICA and ECA. Wu et al. [4], Ricci et al. [16], and Feroci et al. [28] also reported similar and non-significant mean operative times for ICA and ECA groups [4,16,28]. The time difference between the amount of mobilization required for the ECA and the time needed for the anastomosis formation in the ICA is equivocal, thus resulting in no significant operative time difference [28]. Conversely, ICA is deemed more technically challenging because it requires intracorporeal stitches and a more extensive mobilization to create the anastomosis [24]. This study can argue that the laparoscopic skills from various other minimally invasive operations likely translate to ICA; therefore, the learning curve of ICA was less steep, resulting in nonsignificant operative time. Interestingly, Jamali et al. [8] performed a subgroup analysis between operator experiences and found that the level of laparoscopic experiences between surgeons and the learning curve required were not significant contributing factors to the increased operative time. 

LoHS observed in this study was shorter than previously reported in published literature [12,20,22,26,27]. This study also found that the ICA group had a significantly shorter length of stay. An explanation for this phenomenon is that laparoscopic surgery is associated with less postoperative pain and, therefore, early mobilization than open surgery. Generally, the ICA extraction site is smaller than that of ECA, which often translates to less pain. The earlier return to bowel function and a lower morbidity rate in this study group also likely contributed to the shorter hospital stay. 

It has been suggested that ICA is associated with better oncological outcomes due to higher lymph node yield secondary to a higher vascular ligation [6,20]. However, like multiple previous published literature, [4,22,23,29,30]. This study did not find a significant difference in lymph node yield between ICA and ECA. This early outcome study did not evaluate oncological recurrence. Encouragingly, similar published studies have reported no significant difference in locoregional recurrence between the two anastomosis types [4,22,23,29,30].

Limitations

Limitations of this paper include its retrospective design and associated data collection issues, notably the reason and choice of anastomosis selection. This inherently introduces selection bias, as the laparoscopic anastomotic technique was at each surgeon’s discretion. Moreover, the data collated for this study was comprehensive and from a single tertiary teaching referral center for minimally invasive colorectal surgery staffed by CSSANZ fellowship-trained specialists. The follow-up study will be of great interest to see if our findings correlate with other colorectal units in various tertiary hospitals in Australia. The other limitation of this study is its narrow scope, which, as intended, was to look at early center experiences; thus, long-term follow-up is required to report on five-year outcomes, which the authorship team will report on using a planned multi-center prospectively maintained database of long-term clinical and patient-reported outcomes. 

## Conclusions

This early single-center experience showed that ICA is associated with a quicker return to normal bowel function and low morbidity outcomes. ICA participants were positively associated with clinically relevant and health economics outcomes of shorter hospital stays without significantly adding to the procedure's duration times or compromising principles of oncological resection yield.
